# A skin abscess model for teaching incision and drainage procedures

**DOI:** 10.1186/1472-6920-8-38

**Published:** 2008-07-03

**Authors:** Michael T Fitch, David E Manthey, Henderson D McGinnis, Bret A Nicks, Manoj Pariyadath

**Affiliations:** 1 Emergency Medicine Educational Research and Development Group, Department of Emergency Medicine, Wake Forest University School of Medicine, Winston-Salem, North Carolina, USA

## Abstract

**Background:**

Skin and soft tissue infections are increasingly prevalent clinical problems, and it is important for health care practitioners to be well trained in how to treat skin abscesses. A realistic model of abscess incision and drainage will allow trainees to learn and practice this basic physician procedure.

**Methods:**

We developed a realistic model of skin abscess formation to demonstrate the technique of incision and drainage for educational purposes. The creation of this model is described in detail in this report.

**Results:**

This model has been successfully used to develop and disseminate a multimedia video production for teaching this medical procedure. Clinical faculty and resident physicians find this model to be a realistic method for demonstrating abscess incision and drainage.

**Conclusion:**

This manuscript provides a detailed description of our model of abscess incision and drainage for medical education. Clinical educators can incorporate this model into skills labs or demonstrations for teaching this basic procedure.

## Background

Skin abscesses are commonly encountered clinical problems, and the incision and drainage of an abscess is a treatment that is often performed in both emergency and outpatient practice settings. The goal of this project was to develop a model of abscess incision and drainage for educating clinicians about this minor surgical procedure. The importance of educational models for this procedure is evident in the recent resurgence of skin infections and abscesses associated with community acquired methicillin-resistant *Staphylococcus aureus *infections.[1,2] While antibiotic resistance is a growing concern in the treatment of cutaneous infections, simple techniques such as incision and drainage of a skin abscess allow successful treatment of most of these infections without the need for antibiotic therapy. [3-5]

## Results

### Development of the abscess model

Our abscess model was developed in the human anatomy lab at our medical school using a donated human cadaver. The use of fresh cadaveric specimens without traditional preservatives allowed manipulation of dermal structures in a life-like fashion. Areas of erythema on the skin were often already present but were also simulated by application of a lipstick moulage.

Tapioca pudding was diluted 30% with water to simulate purulent material for the abscess contents. Tapioca pudding was the appropriate consistency and color with individual particles to accurately simulate purulent abscess contents. Initial attempts to create a subcutaneous abscess with this simulated purulent material were unsuccessful as intradermal or subcutaneous injections rapidly diffused under the skin and did not create localized collections to correctly simulate an abscess.

A Word catheter (a product typically used in the treatment of Bartholin abscesses) [6], was ideal for creating a contained collection that accurately represented an abscess. The Word catheter was over-filled with about 10 mL of simulated purulent material to represent a contained abscess. When incised with a scalpel, the synthetic material of the Word catheter mimicked the tactile sensation of incising the wall of an abscess. Maintaining constant pressure on an attached syringe filled with additional simulated purulence allowed for a continuous effluent of purulent material when the simulated abscess ruptured upon incision.

**Figure 1:**
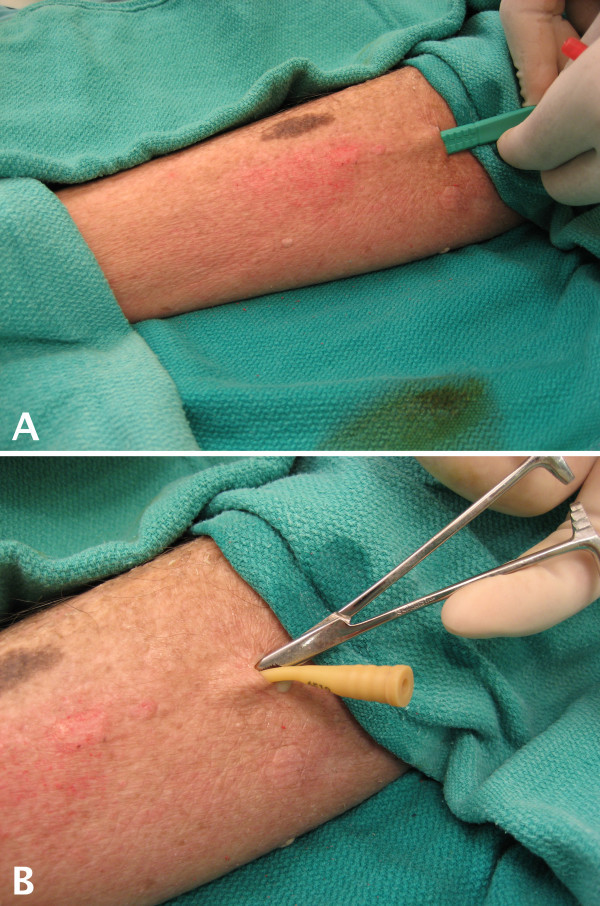
**Initiating the Abscess Model**. A simulated abscess is created on the lateral leg of a fresh cadaveric specimen. A) A scalpel is used to make a skin incision and a subcutaneous tunnel to begin the creation of the abscess model. B) A Word catheter is inserted into the subcutaneous tissues.

**Figure 2:**
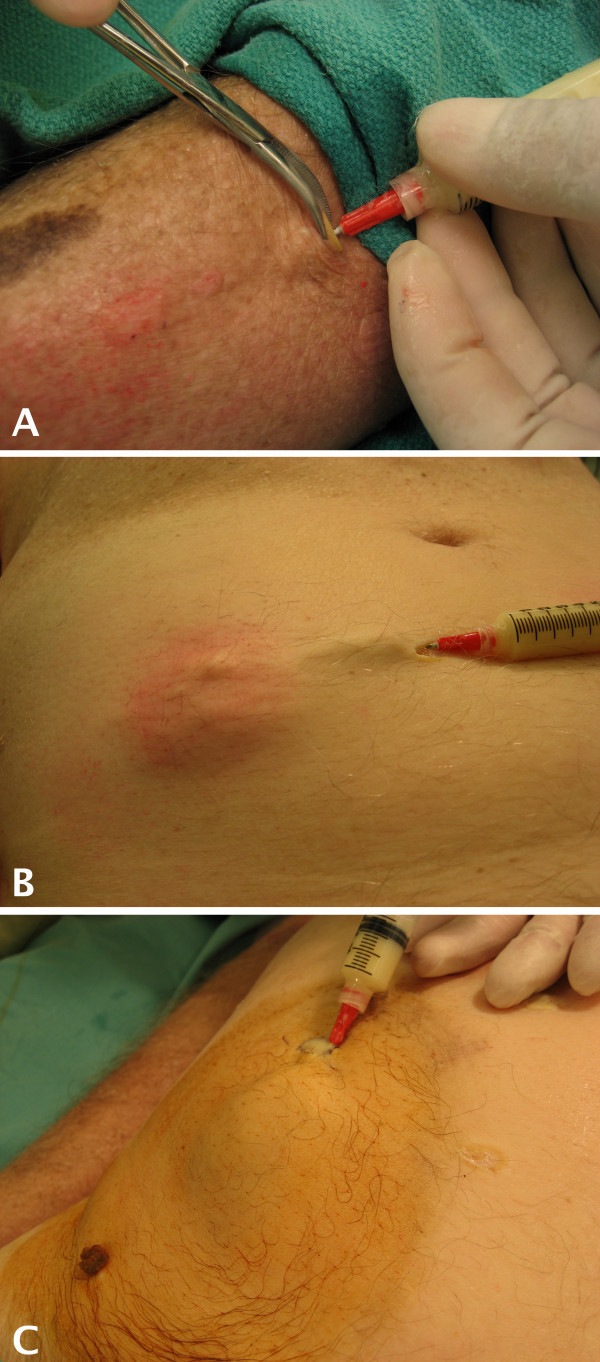
**Injection of Simulated Abscess Material**. The simulated abscess material is manually injected using a hand-held syringe. A) After the Word catheter is completely under the skin, a syringe containing the simulated purulent material is connected. B) Filling of the Word catheter creates a life-like subcutaneous abscess of the abdominal wall in a cadaveric specimen. C) Increasing the quantity and filling pressure allows the operator to create abscesses of various sizes, demonstrated here on the lateral abdominal wall.

This filled catheter was surgically implanted into the cadaveric skin *in situ *using subcutaneous tunneling from a distant incision site [see Figure 1, Panel A]. A scalpel was used to make a skin incision and a tunnel was created under the skin by blunt dissection with hemostats to accept the empty Word catheter [see Figure 1, Panel B]. This allowed the skin overlying the simulated abscess site to remain intact. After placement, the catheter was filled with simulated purulent material using a syringe as described above [see Figure 2]. Filling the Word catheter after placement allowed for realistic displacement and distention of the overlying tissues.

**Figure 3:**
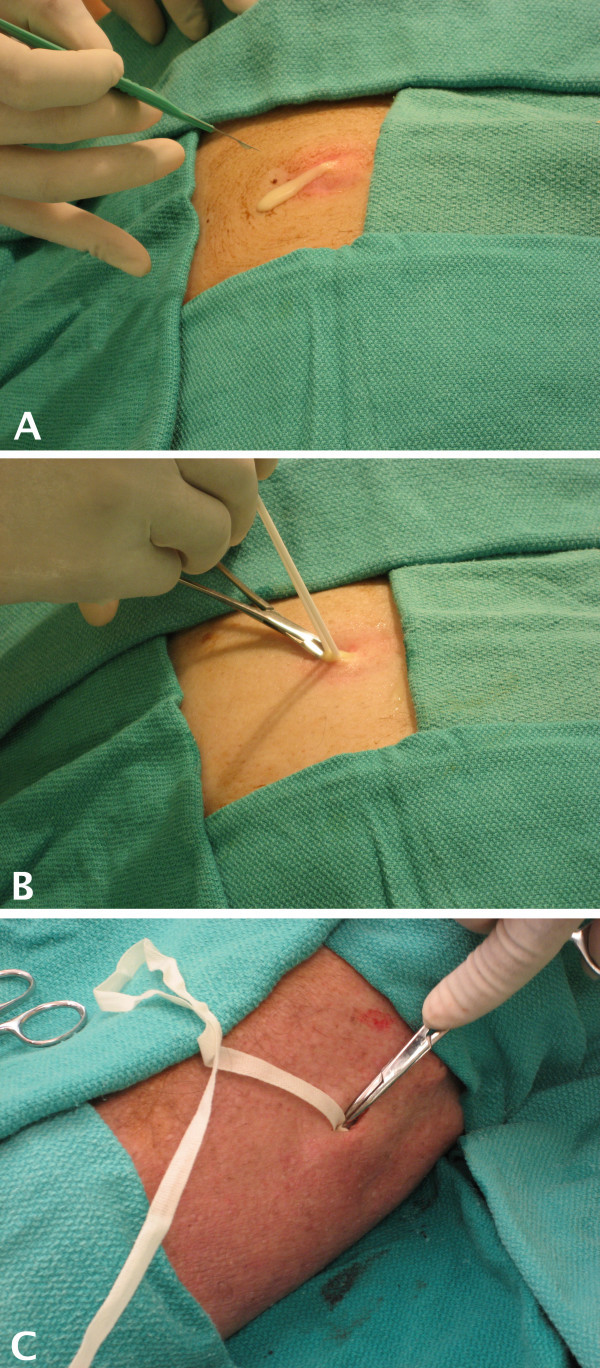
**Using the Abscess Model for Incision and Drainage**. The completed abscess model is ready for incision and drainage. A) A scalpel is used to incise the simulated abscess of the abdominal wall, which leads to realistic drainage of the abscess contents. B) Hemostats and a culture swab are used to sample the interior of the abscess cavity. C) A simulated abscess cavity of the lower leg is filled with wound packing material to complete the incision and drainage procedure.

The size and firmness of the abscess was varied by changing the amount of simulated purulent material that was injected into the catheter, and this created a realistic model ready for an incision and drainage procedure. Draping of the area allowed for concealment of the incision site and the terminal end of the Word catheter to enhance the realistic appearance of the model, and the model was then used to practice incision and drainage [see Figure 3]. The operator was able to adjust the degree of tension of the simulated abscess by altering the force with which the material was injected into the Word catheter. Independent evaluation of the site by visualization and palpation verified the appropriate representation of an abscess.

### Evaluation of the abscess model

Digital video and still photography was used to demonstrate appropriate techniques for the incision and drainage procedure [see Figure 3]. Simulated abscesses were created on the abdomen, leg, and arm of the cadaveric specimen with good results. Figures 1 and 2 contain representative photographs illustrating the creation of the abscess model. Incision of the simulated abscess with a scalpel allowed realistic drainage of the abscess contents [see Figure 3, Panel A). Using culture swabs to obtain a sample of the interior of the abscess [see Figure 3, Panel B] was demonstrated, and the abscess cavity was packed using wound packing material to complete the procedure [see Figure 3, Panel B].

The effectiveness of this model as a realistic way to demonstrate key components of the incision and drainage procedure was evaluated by creating a multimedia video presentation using this model.[7] This video is being used for clinical instruction and has been peer-reviewed and published, and is available on-line to individual subscribers, institutional subscribers, or for free trial subscribers.[7] Feedback obtained from 11 faculty physicians and 12 resident physicians experiencing this model in person and/or video found that all 23 physicians felt it was a realistic way to demonstrate incision and drainage techniques.

## Conclusion

This project developed a model for incision and drainage of skin abscesses to teach clinicians this important clinical procedure. This model is realistic, which is demonstrated by physician review and the use of this technique to create a peer-reviewed and published teaching video.[7] Teaching models like this one are important for educating clinicians without placing real patients at risk for complications of an invasive procedure. A search of the Medline database revealed one other published model which used white glue injected under chicken skin without detailed instructions or associated photographic or video imagery to allow readers to evaluate its fidelity in representing actual skin abscesses.[8] Our own trials demonstrated that injection of simulated abscess material under the skin without containment in the Word catheter was not realistic.

While the realism of our model is evident in the video and still photographs that have been produced using these methods,[7] use of this model as an educational tool has yet to be directly compared to bedside teaching on a live patient. Its use in hands-on teaching sessions will allow further evaluation of whether similar positive results can be achieved for learners when compared to bedside teaching involving real abscesses and live patients. Cadaveric donations could be less available in some areas or the cost of using them may exceed that of other less realistic options for teaching this procedure. However, models such as this one have the added benefit of improving patient safety by providing educational opportunities for medical students, resident physicians, mid-level providers, and practicing physicians before performing incision and drainage on patients presenting with skin abscesses.

## Competing interests

The authors declare that they have no competing interests.

## Authors' contributions

All authors participated in the conception, design, and implementation of the abscess model. MTF drafted the manuscript and all authors participated in the writing and revising of the manuscript. All authors read and approved the final manuscript.

## Pre-publication history

The pre-publication history for this paper can be accessed here:


